# A combined *in silico* and *in vitro* study on mouse Serpina1a antitrypsin-deficiency mutants

**DOI:** 10.1038/s41598-019-44043-3

**Published:** 2019-05-16

**Authors:** Reto Eggenschwiler, Atanas Patronov, Jan Hegermann, Mariane Fráguas-Eggenschwiler, Guangming Wu, Leon Cortnumme, Matthias Ochs, Iris Antes, Tobias Cantz

**Affiliations:** 1 Research Group Translational Hepatology and Stem Cell Biology, Cluster of Excellence REBIRTH, Hannover Medical School, Hannover, 30625 Germany; 2 Department of Gastroenterology, Hepatology and Endocrinology, Hannover Medical School, Hannover, 30625 Germany; 3 Protein Modelling Group, Department of Life Sciences, Technical University Munich, Freising, 85354 Germany; 4 TUM School of Life Sciences, Center for Integrated Protein Science (CIPSM), Technical University Munich, Freising, 85354 Germany; 5 Research Core Unit Electron Microscopy, Hannover Medical School, Hannover, 30625 Germany; 6 Institute of Functional and Applied Anatomy, Hannover Medical School, Hannover, 30625 Germany; 7 Imaging Platform of the Cluster of Excellence REBIRTH, Hannover Medical School, Hannover, 30625 Germany; 8 TWINCORE, Centre for Experimental and Clinical Infection Research, Hannover, 30625 Germany; 9 Max Planck Institute for Molecular Biomedicine, Cell and Developmental Biology, Münster, 48149 Germany; 10 Institute of Vegetative Anatomy Charité - Universitaetsmedizin Berlin, Berlin, 10115 Germany

**Keywords:** Animal disease models, Genetic models, Endoplasmic reticulum

## Abstract

Certain point-mutations in the human *SERPINA1*-gene can cause severe α1-antitrypsin-deficiency (A1AT-D). Affected individuals can suffer from loss-of-function lung-disease and from gain-of-function liver-disease phenotypes. However, age of onset and severity of clinical appearance is heterogeneous amongst carriers, suggesting involvement of additional genetic and environmental factors. The generation of authentic A1AT-D mouse-models has been hampered by the complexity of the mouse *Serpina1*-gene locus and a model with concurrent lung and liver-disease is still missing. Here, we investigate point-mutations in the mouse Serpina1a antitrypsin-orthologue, which are homolog-equivalent to ones known to cause severe A1AT-D in human. We combine *in silico* and *in vitro* methods and we find that analyzed mutations do introduce potential disease-causing properties into Serpina1a. Finally, we show that introduction of the King’s-mutation causes inactivation of neutrophil elastase inhibitory-function in both, mouse and human antitrypsin, while the mouse Z-mutant retains activity. This work paves the path to generation of better A1AT-D mouse-models.

## Introduction

Alpha-1-antitrypsin (A1AT) is an acute phase serine proteinase inhibitor, which is synthesized and secreted as a glycoprotein by hepatocytes and alveolar macrophages in human in order to protect tissue from degradation by endogenous neutrophil elastase (NE). Severe A1AT deficiency is a congenital disorder, which occurs due to certain missense point mutations in the serum protease inhibitor, member 1 (*SERPINA1*) gene. The most predominant of these mutations is the Z-mutation, accounting for >95% of clinical A1AT deficiency cases^1^. This point mutation results in substitution of glutamic acid at position 342 with lysine (E342K), which destabilizes the adjacent reactive centre loop (RCL). Z-A1AT protein is prone to self-aggregation and multimerization, leading to accumulation within the endoplasmic reticulum (ER) of hepatocytes, resulting in inefficient A1AT secretion and low serum concentration in ZZ homozygous individuals. ZZ serum levels are only 10–15% of normal individuals who bear two healthy M-A1AT alleles, which is not sufficient to protect elastin fibers in the lung from breakdown by NE, resulting in chronic obstructive pulmonary disease (COPD). Moreover, the accumulation of Z-A1AT aggregates in hepatocytes can lead to neonatal hepatitis, liver cirrhosis and hepatocarcinoma^2^. There is no cure for the lung or liver disease till date, except for orthotopic organ transplantation. Interestingly, there is considerable variability between ZZ individuals with respect to age of onset and severity of both, pulmonary and hepatic disease. This suggests that other genetic and environmental modifiers might influence the course of the disease^3,4^. Investigation of extended disease mechanisms and research for effective therapeutic approaches are still hampered by the lack of an authentic mouse model for A1AT deficiency. A model for the liver disease created by Carlson and colleagues termed “PiZ-mouse” shows increased necrosis of hepatocytes, inflammation of hepatic tissue and liver fibrosis, which partially recapitulates the more severe liver phenotype in human patients^5^. More recently, full knockout of five *Serpina1a-e* genes created a mouse with lung phenotype^6^. However, potential gain-of-function and loss-of-function interplay can only be investigated when both features are combined within the same animal. Thus, an ‘all-inclusive’ mouse model for the study of severe A1AT deficiency is still missing. Here, we propose the targeted mutation of the endogenous mouse *Serpina1a* gene. We investigated analogous mutations to those known to cause severe A1AT deficiency in human in mouse Serpina1a and analyzed protein destabilization, multimerization and aggregation inside the ER of producer cells using *in silico* and *in vitro* methods. In more detail, we have analyzed homolog equivalent mutants to human Z-A1AT and King’s A1AT, and we demonstrate that both mutations cause multimerization and ER-aggregation of Serpina1a, comparable to their human counterparts. Moreover, to our knowledge, we first show that the King’s mutation functionally inactivates NE inhibition properties of both, human and mouse antitrypsin, whereas the mouse Z-mutant retains activity. Both potential mutation sites are located within close distance on *Serpina1a* Exon V in the mouse genome and should therefore be amenable for targeted genome engineering approaches.

## Results

### *In silico* homology modeling of mouse Serpina1a and assessment of point mutations

**Figure 1:**
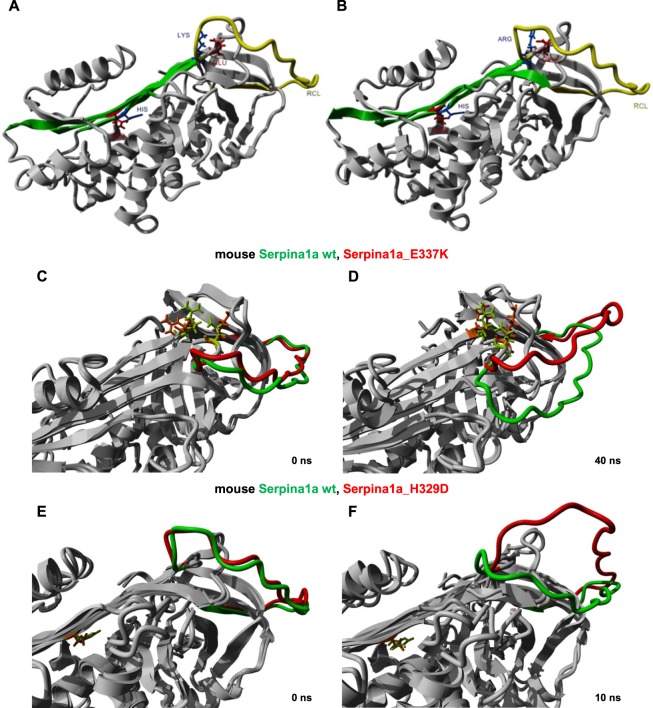
Homology modeling and molecular dynamics (MD) simulation of mouse Serpina1a. (**A**) Crystal structure of human A1AT (PDB-file 1QLP) and (**B**) *in silico* homology model of mouse Serpina1a, with highlighted β-sheet A (green) and RCL (yellow). Positive and negative polar residues forming stabilizing non-covalent bonds at the sites of the Z-mutation (human: Glu342-Lys290; mouse: Glu337-Arg284) and the King’s mutation (human: His334-Asn186; mouse: His329-Asn180) are shown in blue and red, respectively. (**C,D**) RCLs of wild type (green) and E337K mutant (red) Serpina1a at the start and after 40 ns of MD simulation. Similar distortion of RCL was found after 10 ns MD simulation when introducing the H329D point mutation (**E,F**). Amino acid residues involved in non-covalent bond formation at mutation sites are highlighted in light green (wt) and orange (mut) in MD simulation pictures.

The modification of Serpina1a into a secretion-deficient mutant is a potential approach for the generation of authentic A1AT-D mouse models. Rational design of a secretion-deficient mouse antitrypsin variant can be assisted by *in silico* tools such as stability prediction software and molecular dynamics (MD) simulation. However, this requires a 3D model of the protein in question and the 3D crystal structure of mouse Serpina1a has not been determined to date. Therefore, we performed homology modeling based on the known crystal structure of human A1AT. Briefly, alignment of mouse Serpina1a with human A1AT showed a 64.3% pairwise identity and analysis of hydrophobicity and isoelectric points of separate amino acids revealed largely identical set-up of both proteins (Supplementary Fig. S1). Homology modeling was performed based on the human A1AT PDB file 1QLP (Fig. 1A)^7^, as described in methods section and the resultant 3D homology model (hm) of mouse Serpina1a is depicted in Fig. 1B. PDB-files of Serpina1a-hm and 1OLP of human A1AT were then used for *in silico* analysis of point mutations which have been described to cause A1AT deficiency in human^2,8–10^. Only point mutations at amino acids conserved between human and mouse proteins were considered. To this end, open-source tools PoPMuSiC and HoTMuSiC (https://soft.dezyme.com/) were employed for prediction of changes in Gibb’s free energy (ΔΔG) and melting temperature (Tm; Table 1)^11^. A positive value for ΔΔG and a negative value for Tm indicate that the mutation may destabilize the protein. With the exception of P(duarte), all analyzed point mutations were predicted to cause similar changes in protein stability in mouse and human antitrypsin. Notably, introduction of the King’s point mutation (human H334D, mouse H329D) was predicted to cause the strongest destabilization, comparable to Null(ludwigshafen), which was reported to distort tertiary structure in human A1AT and classifies as a dysfunctional allele^12^. Moreover and importantly, the Z point mutation (human E342K, mouse E337K) was predicted to destabilize mouse Serpina1a as well (Table 1). Based on these findings, we decided to employ molecular dynamics (MD) simulation for analysis of distortion of the antitrypsin RCL, which is a pre-requisite for aggregate formation of human A1AT^13^. To this end, we focused on the clinically highly relevant Z mutation, and the highly destabilizing King’s mutation for MD analysis. As depicted in in Fig. 1C–F, Z and King’s mutations both severely distorted RCL of Serpina1a, after 40 ns and 10 ns of simulation, respectively. We found similar results for human A1AT as well (Supplementary Fig. S2A–D). A detailed description of MD simulation can be found in the methods section.

**Table 1:** Prediction of Gibb’s free energy and melting temperature changes in antitrypsin mutants.

mutation name	residue change	ΔΔG (kcal/mol)		ΔTm (°K)	
	human/*mouse*	human A1AT, PDB-ID: 1QLP	mouse Serpina1a hm	human A1AT, PDB-ID: 1QLP	mouse Serpina1a hm
S(iiyama)	S53F/*S47F*	−0.01	−0.13	−0.86	−0.2
Null(ludwigshafen)	I92N/*I86N*	2.8	2.68	−4.98	−4.17
P(duarte)	D256V/*D250V*	−0.36	0.26	0.1	−1.03
S	E264V/*E258V*	0.11	0.91	−0.93	−1.8
King’s	H334D/*H329D*	3.12	3.02	−5.32	−5.22
W(bethesda)	A336T/*A331T*	0.76	0.74	−1.61	−1.75
Z	E342K/*E337K*	0.82	0.76	−1.51	−0.63
M(heerlen)	P369L/*P364L*	0.49	0.62	−2	−2.08

Difference of Gibb’s free energy (ΔΔG) and change in melting temperature (Tm) were calculated employing PoPMuSiC and HoTMuSiC software using the PDB-file 1QLP for human A1AT and the homology model (hm) for mouse Serpina1a.

### The Z and King’s mutations both cause aggregation and ER accumulation of Serpina1a

**Figure 2:**
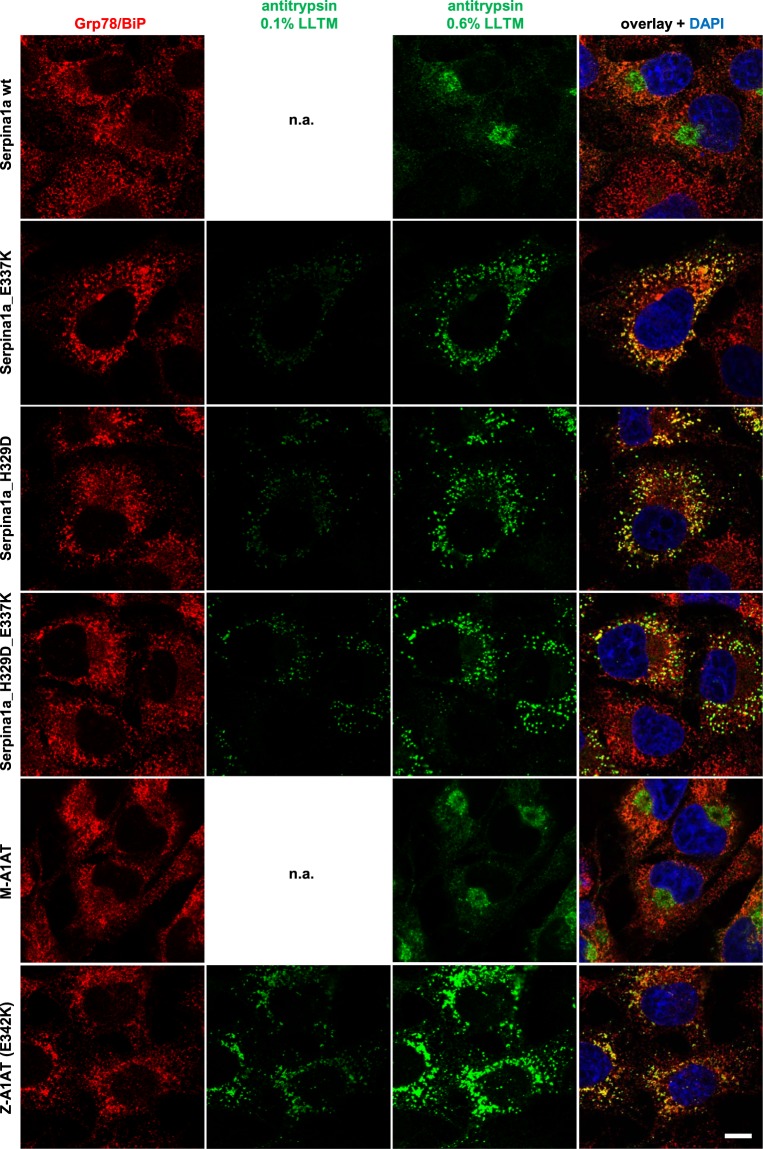
Serpina1a mutants imitate intracellular distribution of human Z-A1AT. Confocal laser immunofluorescence analysis of COS-7 cells expressing wild type (wt; top row), E337K, H329D or H329D_E337K double mutant Serpina1a (second to fourth row), compared to cells overexpressing normal human M-A1AT or E342K Z-A1AT (fifth and sixth row). Mouse Serpina1a and human A1AT were stained with Alexa Fluor 568 secondary antibody (green) and exposed to 0.6% laser light transmission (LLTM). Mutant-expressing cells were additionally exposed to 0.1% LLTM, as the very strong signal resulted in over-saturation at 0.6%. ER-marker Grp78/BiP was stained with Alexa Fluor 647 secondary antibody (red) and cell nucleus was stained using DAPI (blue). Scale bar: 10 µm.

**Figure 3:**
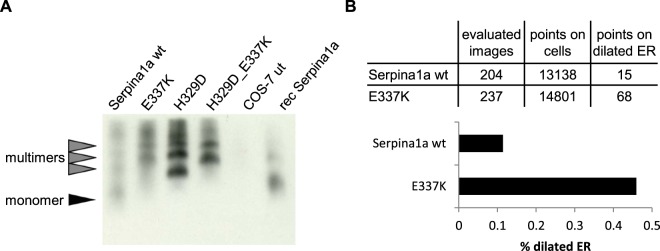
Serpina1 mutants self-aggregate and cause ER dilation. (**A**) Native western blot with cell lysates from COS-7 cells expressing wt and mutant Serpina1a. Untransduced COS-7 cells and recombinant Serpina1a served as negative and positive controls, respectively. (**B**) Quantification of dilated ER in electron microscopy images from wild type Serpina1a and E337K-mutant overexpressing COS-7 cells. Over 27,000 points were analyzed in >400 non-overlapping images and percentage of points in dilated ER was calculated.

As demonstrated above, *in silico* analysis predicted destabilization and RCL-distortion for Z and King’s mutations, in both human and mouse antitrypsin. To investigate whether these mutations can lead to aggregation of mouse Serpina1a in cells *in vitro*, we employed a previously published lentiviral expression vector encompassing a Serpina1a CDS followed by an IRES and an eGFP-T2A-puroR cassette^14^ (Supplementary Fig. S3A), and introduced the King’s (H329D) and Z (E337K) mutations each separately or combined (H329D_E337K) by site-directed mutagenesis. Lentiviral vector particles with different Serpina1a mutants were transduced into COS-7 African green monkey kidney cells, a frequently utilized cellular model for studies of aggregation of human A1AT, which was recently also employed for functional studies of mouse antitrypsin paralogs^8,14^. Transduced cells were selected using puromycin and analyzed by flow cytometry for comparable expression of eGFP marker gene (Supplementary Fig. S3B). Bulk populations stably expressing wild type (wt) or mutant Serpina1a were first characterized using confocal laser immunofluorescence microscopy. Signal of wild type Serpina1a was found to be co-localized with ER-marker Grp78/BiP, but was most dense in the Grp78/BiP negative region, which are most likely Golgi structures (top row of Fig. 2, staining controls in Supplementary Fig. S3C). On the contrary, in H329D, E337K and H329D_E337K mutant expressing cells the strongest signal for Serpina1a coincided with Grp78/BiP (Fig. 2). Moreover, the overall distribution of mutant proteins looked very tuberous, a striking morphological difference compared to wt Serpina1a. We detected a comparable intracellular distribution when overexpressing human E342K Z-A1AT while normal human M-A1AT was similar to wt Serpina1a (Fig. 2). Human Z-A1AT polymers can readily be detected by native western blotting^15^, and therefore we investigated if this method could be applied to visualize multimers of Serpina1a mutant proteins as well. As shown in Fig. 3A, lysates from cells expressing wild type Serpina1a mostly showed monomeric protein, comparable to purified recombinant Serpina1a. In all analyzed mutant proteins, monomer band was absent and multimer bands were prominent (see also Supplementary Fig. S4). This was also confirmed when overexpressing the same constructs in the human hepatocarcinoma cell line HepG2 (Supplementary Fig. S5A). The slightly higher band of recombinant Serpina1a results from the 24 amino acid ER-signal peptide which is not cleaved off when expressing the protein in bacteria, as detected when analyzing the same samples using denaturing SDS-PAGE western blot (Supplementary Fig. S5B). The apparent lower size of H329D mutant multimers in native blots may be explained by its reduced isoelectric point as consequence of exchanging positively charged histidine with negatively charged aspartic acid (Supplementary Fig. S5C). Finally, it is known that expression of human Z-A1AT results in ER-dilation of affected hepatocytes and aggregation of mutant human A1AT can be detected by electron microscopy using gold-labeled antibodies^16^. Thus, we employed transmission electron microscopy (TEM) with immunogold labelling for analysis of COS-7 cells expressing wt Serpina1a or E337K mutant. Dilation of ER was observed in both samples, but was considerably increased in E337K-expressing cells (Fig. 3B; Supplementary Fig. S6). Immunoreaction of antibodies directed against Serpina1a was clearly discernable in E337K samples in intracellular membrane-surrounded compartments resembling ER (Supplementary Fig. S6F–J). In wt Serpina1a-expressing cells, such compartments were also observed, but only seldom showed positive antibody binding (Supplementary Fig. S6A–E), which would be expected, as normal Serpina1a should not accumulate within the ER.

### The King’s mutation functionally inactivates human A1AT and mouse Serpina1a

**Figure 4:**
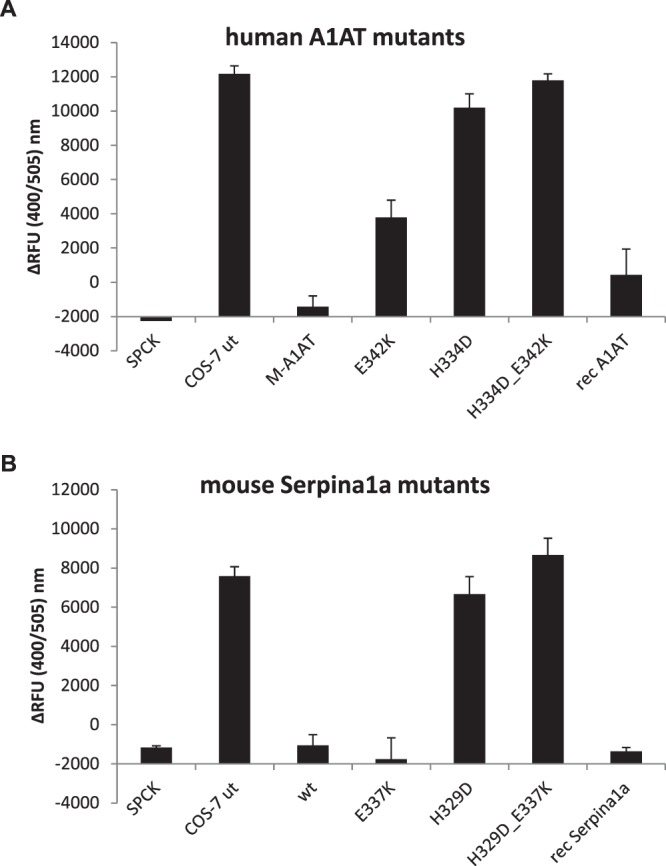
The King’s mutation functionally inactivates human A1AT and mouse Serpina1a. Analysis of neutrophil elastase (NE) inhibitory function of supernatants from human A1AT (**A**) or mouse Serpina1a (**B**) expressing COS-7 cells. Human and mouse antitrypsin protein concentrations were determined by western blot quantification (see Supplementary Fig. S6A–D) and 10 ng of total protein were used for inhibition of NE. Recombinant human A1AT or mouse Serpina1a protein was included for standardization, whereas chemical NE-inhibitor SPCK and supernatants from untransduced COS-7 cells served as positive and negative controls, respectively. ΔRFU was calculated as difference in fluorescence at Ex/Em = 400/505 nm between time points t = 0 min and t = 30 min. All supernatants were analyzed using n = 3 biological replicates × n = 3 technical replicates each. SPCK and recombinant protein controls were analyzed using n = 3 technical replicates. Error bars represent +SD. A full statistical evaluation using one-way ANOVA can be found in Supplementary Table 2.

Some mutations are known to distort the structure of human A1AT severely, leading to abrogation of proteinase inhibitory function and qualifying the dysfunctional allele as ‘Null’, similar to a gene-knockout^9^. *In silico* tools predicted that the King’s mutation introduces a strong destabilization, similar to Null(ludwigshafen) (Table 1). Therefore, we analyzed whether the Z- or the King’s mutation would cause functional defects in mouse Serpina1a. To this end, COS-7 cells overexpressing mouse Serpina1a or human A1AT variants were cultivated in serum-free medium and supernatants were harvested after 48 h and quantified by western blot analysis (Supplementary Fig. S7A–D). Supernatants were diluted to equal protein concentration and analyzed for functional inhibition of NE using a fluorescence-based assay (Fig. 4A,B). The Z mutation slightly decreased the functionality of human A1AT, but not mouse Serpina1a, whereas the King’s mutation led to functional inactivation in both, human A1AT and mouse Serpina1a. Notably, double mutants of both, human and mouse antitrypsin functionally inactivated the proteins as well and their NE-inhibitory activities were non-distinguishable from those of negative controls (COS-7 ut).

## Discussion

Severe A1AT-deficiency can cause chronic emphysema and liver damage in human patients. To date, no authentic all-inclusive mouse models are available for extensive *in vivo* investigation of disease mechanisms and for evaluation of novel treatment strategies, such as precision genome engineering-assisted correction of disease phenotype. Here, we propose the introduction of homolog equivalent mutations known to cause severe A1AT deficiency in human into the mouse *Serpina1a* gene. In contrast to humans, mice harbor 3–5 antitrypsin genes (depending on the exact mouse strain), of which *Serpina1a* has earlier been reported to be essential, as its knockout resulted in embryonic lethality in the 129/SvJ mouse strain^17^. *Serpina1b* knockout in 129-strain derived mouse ES cells resulted in strong distortion of Mendelian inheritance pattern, suggesting that this gene may have vital functions as well which eventually was partially rescued by Serpina1a expression^18^. Recently, these findings have been challenged by a report about the creation of viable C57BL/6J *Serpina1a-e* knockout mice^6^. While the quintuple knockout lung phenotype is convincing, a controversial discussion with previous findings is still missing. It is known that gene knockouts can have different consequences regarding viability in different mouse strains^19–25^. Thus, backcrossing of *Serpina1a-e* knockout mice could reveal whether vital functions of *Serpina1a* and *Serpina1b* could be strain-dependent. An aggregation-prone yet functional mutant allele, such as *Serpina1a_E337K* may therefore be a more attractive choice for generation of A1AT deficiency models from certain mouse strains than a full knockout.

Interplay of gain-of-function and loss-of-function aspects of the disease may only be adequately investigated in a model which features both phenotypes. There is strong indication in current literature that presence of normal, fully functional M-A1AT may help to ameliorate severity of liver disease in human. For instance, human M/Z-A1AT heterozygotes rarely present with a severe liver phenotype, even though Z-A1AT multimers still accumulate in hepatocytes^26,27^. Moreover, A1AT augmentation therapy can have beneficial effects on wound healing in humans, reducing inflammation and preventing tissue damage^28^ and low serum levels of A1AT have been implicated in liver cancer, bladder cancer, gall bladder cancer, malignant lymphoma, and lung cancer^29^. It is also possible that protection of liver tissue by endogenous mouse antitrypsin may ameliorate a more severe liver phenotype in PiZ-mice^5^. Thus, an authentic mouse model for A1AT deficiency would have to show reduced serum activity of proteinase inhibitors but also account for aggregation and ER-retention of mutant antitrypsin in hepatocytes. To this end, others have suggested introduction of the mutant human Z-A1AT gene into *Serpina1a-e* knockout mice^6^: However, it is unclear at this point how tissue-specific expression would be ensured, an issue which was not resolved in PiZ mice, where the human antitrypsin-promoter was used to drive expression of Z-A1AT^30^. Moreover, it has recently been uncovered that NE is a highly complex enzyme which self-maturates by auto-processing and resultant alteration of substrate specificity was proposed to be one of the reasons why attempts to develop specific NE inhibitors have failed in the past^31^. In light of these findings, specificity of interaction of human antitrypsin with mouse NE would have to be confirmed prior to creation of a *Serpina1a-e*^−/−^ Z-A1AT knock-in mouse model. These shortcomings could be overcome by introduction of a disease-causing point mutation into an endogenous mouse antitrypsin gene instead. We have confirmed that Serpina1a mutants show similar behavior with respect to protein aggregation and ER-retention as their human counterparts. There is viable indication that Serpina1a was amenable for mutations with desired effects such as multimerization and ER-aggregation. For instance, several mutations have been described in the gene encoding for neuroserpin *SERPINI1* in familial encephalopathy with neuroserpin inclusion bodies (FENIB) patients, which cause multimerization and ER-accumulation of the protein in neurons^32,33^. Moreover, a homolog equivalent to the human Z-mutation leads to self-aggregation propensity and loss-of-function in the *d. melanogaster* serpin *necrotic*. *Necrotic_E421K* mutants hatch as weak adults that die within a few days of eclosion^34^. Finally, a homolog equivalent to the Z-mutation was described in heparin cofactor II (E428K), which caused a >90% decrease in serum level of this antithrombin serpin^35^. Together, this suggests that multimerization and ER aggregation are inherent features of destabilized serpins and that homolog equivalent mutations can have similar consequences even for distantly related serpins. In our work, introduction of the King’s mutation was predicted to cause the strongest destabilization *in silico* and was found to functionally inactivate NE inhibition of both, mouse Serpina1a and human A1AT. In contrast, human Z-A1AT mutant protein is known to retain partial activity^36^ and we confirm that mouse Serpina1a retains functional activity after introduction of an analogous mutation as well. However, the methods presented here however do not allow final conclusions about relative efficiencies between human and mouse antitrypsin mutants, as the NE-assay relies on recombinant human NE (Uniprot P08246) and specificity of interaction and inhibition efficiency may differ between the two species. Finally, the exact mechanism for Serpina1a aggregation remains yet to be determined, an issue which is still a matter of an ongoing debate and has been difficult to resolve for human Z-A1AT^37–40^. Nevertheless, our data reveals important qualitative aspects regarding introduction of analogous point mutations in mouse Serpina1a, which is an encouraging step towards generation of better mouse models for severe A1AT deficiency.

## Conclusion

In summary, we propose two mutants of mouse Serpina1a, which could be employed for generation of mouse models for severe A1AT deficiency. The King’s mutation would likely lead to a functional knockout of the Serpina1a gene, while an allele with the Z mutation would retain functionality while showing a secretion deficiency phenotype. Both variants could be introduced by single base-pair editing and would result in ER aggregation of Serpina1a inside producer cells. Backcrossing to *Serpina1a-e*^−/−^ mice would generate all-inclusive mouse models, accounting for tissue-specific expression, antitrypsin aggregation and ER-dilation as well as specific interaction of mutant antitrypsin with mouse proteases.

## Methods

### Molecular cloning and PCRs

Molecular cloning of lentiviral expression vectors ‘Lenti CAG-SA1A-I-G2AP’, encompassing a CAG promoter driven Serpina1a CDS followed by IRES and eGFP-T2A-puroR, its ΔNsiI-derivate ‘Lenti CSI-G2AP’ as well as ‘Lenti SF-SA1A-I-GFP’ and ‘Lenti-CG2AP’ was described elsewhere^14,41^. Using Lenti SF-SA1A-I-GFP as PCR template, mutations were then introduced into the Serpina1a CDS by site directed mutagenesis. Primer ‘DOM-1 NsiI rev’ was used together either with ‘D329 for’ or ‘K337 for’ or ‘D329 K337 for’ for introduction of H329D, E337K or H329D+E337K point mutations, respectively. Resultant 246 bp fragments were digested using Kpn2I + NsiI and inserted into Lenti SF-SA1A-I-GFP digested with the same enzymes, resulting in ‘Lenti SF-H329D-I-GFP’, ‘Lenti SF-E337K-I-GFP’ and ‘Lenti SF-H329D-E337K-I-GFP’. These three plasmids were then digested with AgeI + BsrGI and the cassette containing Serpina1a mutant CDS joined to IRES-eGFP was inserted into Lenti CG2AP digested with the same enzymes, resulting in ‘Lenti CAG-H329D-I-G2AP’, ‘Lenti CAG-E337K-I-G2AP’ and ‘Lenti CAG-H329D-E337K-I-G2AP’. For cloning of human A1AT expression constructs, the CDS of human wild type M-A1AT was PCR-amplified using cDNA isolated from the human hepatocarcinoma cell line HepG2 at a final concentration of 1 ng/ul as template and ‘hA1AT cDNA AgeI for’ and ‘hA1AT cDNA NsiI rev’ primers. Resultant 1275 bp amplicon was digested with AgeI + NsiI and inserted into pUC57-Serpina1a^14^ digested with the same enzymes, thereby replacing Serpina1a CDS with human M-A1AT CDS, resulting in ‘pUC57 M-A1AT’. Using pUC57 M-A1AT as PCR template, mutations were then introduced into the A1AT CDS by site directed mutagenesis. Primer ‘hA1AT cDNA AgeI for’ was used together either with ‘H334D AvaI rev’ or ‘E342K AvaI rev’ or ‘H334D E342K AvaI rev’ for introduction of H334D, E342K or H334D+E342K point mutations, respectively. Resultant, 1173 bp PCR amplicons were digested using AvaI + BamHI and inserted into pUC57 M-A1AT digested with the same enzymes, thereby replacing wild type A1AT CDS with mutant sequences, resulting in ‘pUC57 H334D-A1AT’, ‘pUC57 E342K-A1AT’ and ‘pUC57 H334D-E342K-A1AT’. All four pUC57 constructs containing human A1AT variants were then digested using AgeI + NsiI and inserted into Lenti CSI-G2AP digested with the same enzymes, thereby replacing CDS of Serpina1a with those of human A1AT variants, resulting in ‘Lenti CAG-A1AT-I-G2AP’, ‘Lenti CAG-H334D-I-G2AP’, ‘Lenti CAG-E342K-I-G2AP’ and ‘Lenti CAG-H334D-E342K-I-G2AP’. All PCRs were performed using Phusion Hot Start II DNA Polymerase with HF-buffer (Thermo Scientific #F549L) according to manufacturer’s procedures. Melting temperatures of primers were calculated using the Tm calculator on the Thermo Scientific homepage (https://www.thermofisher.com/) and a 6-step gradient PCR was performed from the lowest oligo’s Tm −2 °C to the highest oligo’s Tm + 6 °C in order to determine the optimal annealing temperature yielding specific products. Fidelity of all cloned PCR products was confirmed Sanger Sequencing. Sequences of PCR primers are given in Supplementary Table 1.

### Production and titration of lentiviral vectors

Lentiviral vectors were produced by transient transfection of Lenti CAG-SA1A-I-G2AP, Lenti CAG-H329D-I-G2AP, Lenti CAG-E337K-I-G2AP, Lenti CAG-H329D-E337K-I-G2AP, Lenti CAG-A1AT-I-G2AP, Lenti CAG-H334D-I-G2AP, Lenti CAG-E342K-I-G2AP or Lenti CAG-H334D-E342K-I-G2AP along with pCDNA3.GP.4xC, pMD2.G and pRSV-Rev into HEK293T cells using the CaCl_2_ protocol and titrated using flow cytometry, as described previously^14^.

### Cell lines and transductions

COS-7 African green monkey kidney, HEK293T and HepG2 human hepatoma cells were cultured in DMEM GlutaMax 4.5 g/L glucose supplied with 10% FBS and 1% Pen/Strep. For generation of transgenic cell populations, 100,000 COS-7 cells were transduced at multiplicity of infection (MOI) 30 or and selected for 2 days with 6 µg/ml puromycin and for an additional 3 days with 3 µg/ml. HepG2 cells were transduced at MOI 50 and subjected to the same selection procedure. For NE inhibition assays, COS-7 cells were cultivated for 48 h in serum-free InVitrus medium (Cell Culture Technologies).

### Neutrophil elastase inhibition assay

Inhibition of NE with supernatants of transduced COS-7 cells in serum-free InVitrus medium was analyzed as described previously^14^. Neutrophil Elastase Inhibitor Screening Kit (Abcam #ab118971) was used according to manufacturer’s procedures and progression of substrate conversion was measured on a Tecan Infinite M200 plate reader at Ex/Em = 400/505 nm.

### Immunocytochemistry and confocal microscopy

Immunocytochemistry for mouse Serpina1a and human A1AT was performed as previously described^14,41^. Primary antibodies goat anti-human AAT A80–122 A (Bethyl, Montgomery TX, USA), goat anti-mouse Serpin A1c/α1Antitrypsin (R&D Systems, AF2979) and rabbit anti-GRP78 BiP (Abcam, ab21685) were all used at dilution of 1:1,000. Secondary antibodies Donkey-anti-Goat IgG (H + L) Alexa Fluor® 568 (Thermo Fisher Scientific, #A-11057,) and Donkey-anti-Rabbit IgG (H + L) Alexa Fluor® 647 (Thermo Fisher Scientific, #A-31573) were used at dilution 1:2,000.

Immunofluorescence was imaged with a confocal laser scanning microscope (TCS SP8, LEICA Microsystems, Wetzlar, Germany) using a PL-APO 63x oil-immersion objective, numerical aperture of 1.4. Laser light transmission at 488 nm was set to 0.1%, or 0.6%, accordingly.

### Western blot

SDS-PAGE western blots were performed as previously described^14,41^. Goat anti-human AAT A80–122 A (Bethyl, Montgomery TX, USA) and goat anti-mouse Serpin A1c/α1Antitrypsin (R&D Systems, AF2979) were both used at dilution of 1:1,000. Secondary antibody donkey IgG anti goat IgG (H + L)-HPRO (Dianova #705-035-147) was diluted 1:2,000. Native gel electrophoresis was performed based on a previously published protocol^41^, using a 4–15% gradient Mini-PROTEAN® TGX™ Precast Gel (Biorad #4561084) and same antibodies and dilutions were used as for SDS-PAGE blots.

### Electron microscopy, immunogold labelling and EM quantification

After fixation in 200 mM Hepes buffer, pH 7.35, containing 4% paraformaldehyde and 0.1% glutaraldehyde, samples were infiltrated in 30% glycerol for 2 h and frozen in liquid nitrogen. Freeze substitution was carried out in methanol containing 1% uranyl acetate at −90 °C overnight. After washing in pure methanol, temperature was raised to −40 °C and samples were infiltrated in Lowicryl HM20. Polymerization was done by UV-light at −40 °C for 48 h and at RT for 48 h. 70 nm sections were mounted on copper grids and imaged with post-staining in a Morgagni TEM (FEI) operated at 80 kV.

For immunogold labelling, sections were incubated in the following solutions at room temperature: 5 min in PBST (PBS containing 0.05% Tween-20); 45 min with primary antibody goat anti-mouse Serpin A1c/α1Antitrypsin (R&D Systems, AF2979) diluted 1:20 in PBST; 5 × 5 min in PBST; 20 min with secondary antibody rabbit anti-goat IgG 10 nm gold (BBI Solutions #EM.RAG10) diluted 1: 50 in PBST; 5 × 5 min in PBST; 2 × 10 sec in water; 5 min in 1% aquous uranyl acetate; 2 × 10 sec in water.

For quantification of TEM data about 200 square images of Serpina1a wt and E337K mutant overexpressing COS-7 cells each were recorded at 11 μm edge length by systematic uniform random sampling. A point grid of 361 points was used on every image to count positive hits on cell profiles and profiles of dilated ER, which was judged by the occurrence of ribosomes on the surrounding membrane.

### Homology modeling and molecular dynamics simulation

The X-ray based model of a wild type human A1AT (PDB-ID: 1QLP) was used as a template for homology modeling and subsequent molecular dynamics simulation. In order to model the mutants (human H334D, E342K and mouse H329D, E337K), point mutations were introduced in each structure. The homology modeling was carried out by IRECS^42^ and the inputs for the molecular dynamics were prepared with LEaP12 (http://hincklab.uthscsa.edu/html/soft_packs/amber6/AMBER-sh-5.html).

Molecular dynamics simulations were carried out with Amber-12 using ff12sb force field^43^. Complexes were centered in a solvent box with boundaries located at 15 Å distance from the outermost solute atoms in each direction. Periodic boundary conditions were applied. The boxes were filled with water molecules with the TIP3P water model^44^ and counterions (Na^+^ and Cl^−^) were added. For long range electrostatics Particle Mesh Ewald was used with cutoff of 14 Å. The potential energy of the system was initially minimized. The position restraints of the heavy atoms in the protein were released in two steps: (1) minimization with 2.4 kcal/(mol Å^2^) position restraints on all the heavy atoms in the protein; (2) minimization performed without position restraints. Afterwards, the systems were simulated for 100 ps steps with gradual temperature increase from 0 to 50 K with restraints of 2.4 kcal/(mol Å^2^) on all heavy atoms, from 50 to 200 K with restrains of 2.4 kcal/(mol Å^2^) only on the backbone atoms and from 200 to 300 K with restraints on the backbone atoms with force of 0.24 kcal/(mol Å^2^). Finally, the protein molecules were simulated at a constant temperature (300 K) by using the Berendsen thermostat at default settings in the NPT ensemble for 40 ns in the production run for human and mouse wild type and for human E342K and E337K mutants. Production run for human H334D and mouse H329D was 10 ns. The results were analyzed with VMD^45^ and Yasara View [http://www.yasara.org].

### Statistical methods

All error bars represent positive standard deviation calculated from at least n = 3 independent measurements. Determination of mouse and human antitrypsin protein concentrations in Supplementary Fig. S7C,D was performed using n = 3 collections of supernatants from separate wells. The same supernatants were used for determination of NE-inhibition using n = 3 biological replicates with n = 3 technical replicates each in Fig. 3A,B. A full statistical evaluation of Figs 3A,B and S7C,D using one-way ANOVA with Tukey’s post-test at 95%CI can be found in Supplementary Table 2.

## Supplementary information

Supplementary Dataset 1

## Data Availability

The authors fully comply with the journal’s policy regarding sharing of research materials used in this publication and declare that they are in possession of those materials and that they have the necessary infrastructure for material transfers.
