# Supplementary material for: A combined in silico and in vitro study on mouse Serpina1a antitrypsin-deficiency mutants
Source: Sci Rep. 2019 May 16;9:7486. doi: 10.1038/s41598-019-44043-3 (PMC6522476; doi:10.1038/s41598-019-44043-3)
Supplement: Supplementary file 1 — Supplementary Dataset 1 [file 41598_2019_44043_MOESM1_ESM.pdf]

Supplementary Information

A combined *in silico* and *in vitro* study on mouse Serpina1a antitrypsin-deficiency mutants

Reto Eggenschwiler*,[1,2] Atanas Patronov,[3,4] Jan Hegermann,[5,6,7] Mariane Fráguas-Eggenschwiler,[2,8] Guangming Wu,[9] Leon Cortnumme,[1,2] Matthias Ochs,[5,6,7,10] Iris Antes,[3,4] Tobias Cantz*,[1,2,9]

[1]Research Group Translational Hepatology and Stem Cell Biology, Cluster of Excellence REBIRTH, Hannover Medical School, Hannover, 30625, Germany
[2]Department of Gastroenterology, Hepatology and Endocrinology, Hannover Medical School, Hannover, 30625, Germany
[3]Protein Modelling Group, Department of Life Sciences, Technical University Munich, Freising, 85354, Germany
[4]TUM School of Life Sciences, Center for Integrated Protein Science (CIPSM), Technical University Munich, Freising, 85354, Germany
[5]Research Core Unit Electron Microscopy, Hannover Medical School, Hannover, 30625, Germany
[6]Institute of Functional and Applied Anatomy, Hannover Medical School, Hannover, 30625, Germany
[7]Imaging Platform of the Cluster of Excellence REBIRTH, Hannover Medical School Hannover, 30625, Germany
[8]TWINCORE, Centre for Experimental and Clinical Infection Research, Hannover, 30625, Germany
[9]Max Planck Institute for Molecular Biomedicine, Cell and Developmental Biology, Münster, 48149, Germany
[10]Institute of Vegetative Anatomy Charité - Universitaetsmedizin Berlin, Berlin, 10115, Germany

*__Correspondence:__

Dr. rer. nat. Reto Eggenschwiler, Translational Hepatology and Stem Cell Biology, Cluster of Excellence REBIRTH, Hannover Medical School, 30625 Hannover, Germany
Phone: +49 511/532-5259; e-mail: eggenschwiler.reto@mh-hannover.de

Prof. Dr. med. Tobias Cantz, Translational Hepatology and Stem Cell Biology, Cluster of Excellence REBIRTH, Hannover Medical School, 30625 Hannover, Germany
Phone: +49 511/532-5251; e-mail: cantz.tobias@mh-hannover.de

Figure S1

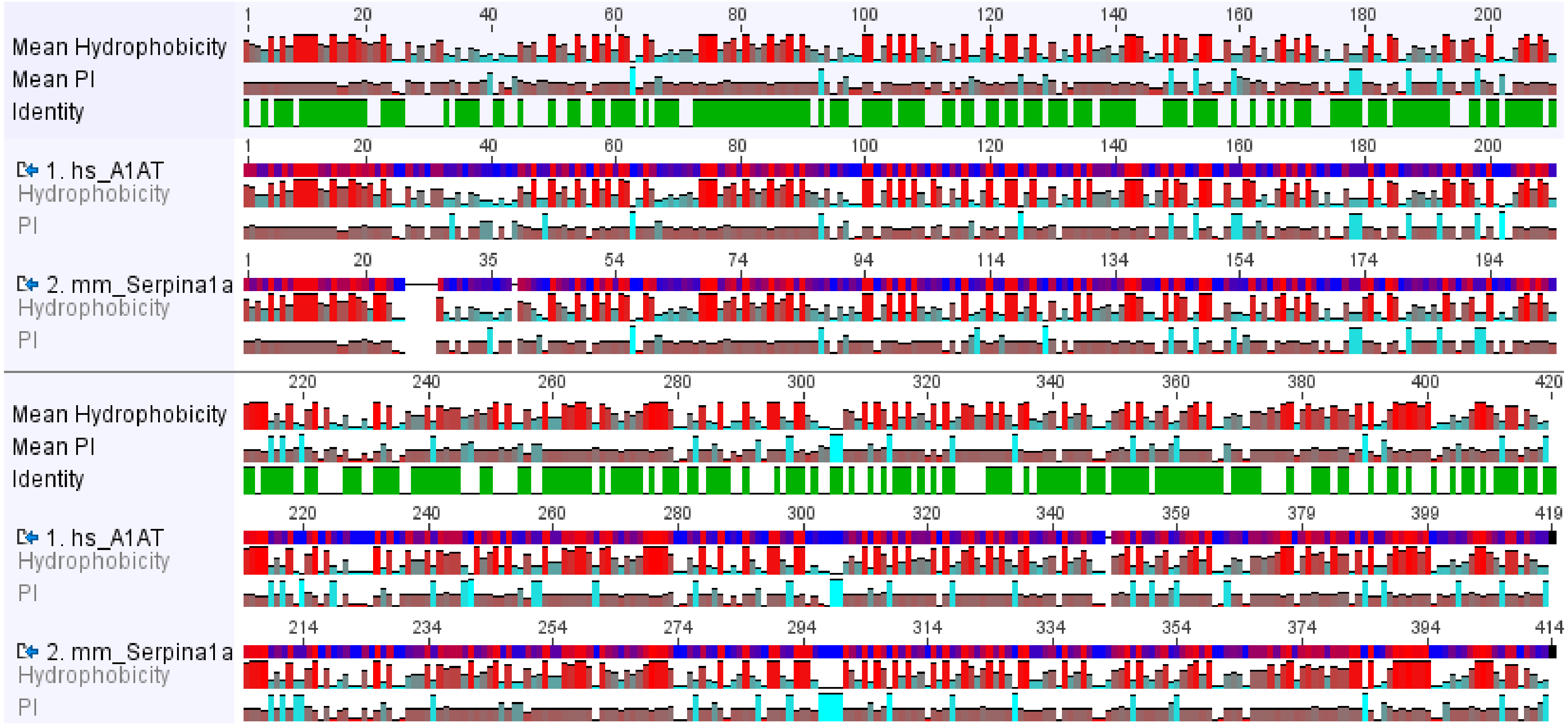

Supplementary Figure S1: Alignment of human and mouse antitrypsin proteins. Amino acid sequences of Human (hs_A1AT) and mouse (mm_Serpina1a) antitrypsin were aligned using Geneious software (Biomatters Ltd.) and hydrophobicity as well as isoelectric point (PI) from all amino acids are shown. Pairwise identity is displayed in green.

Figure S2

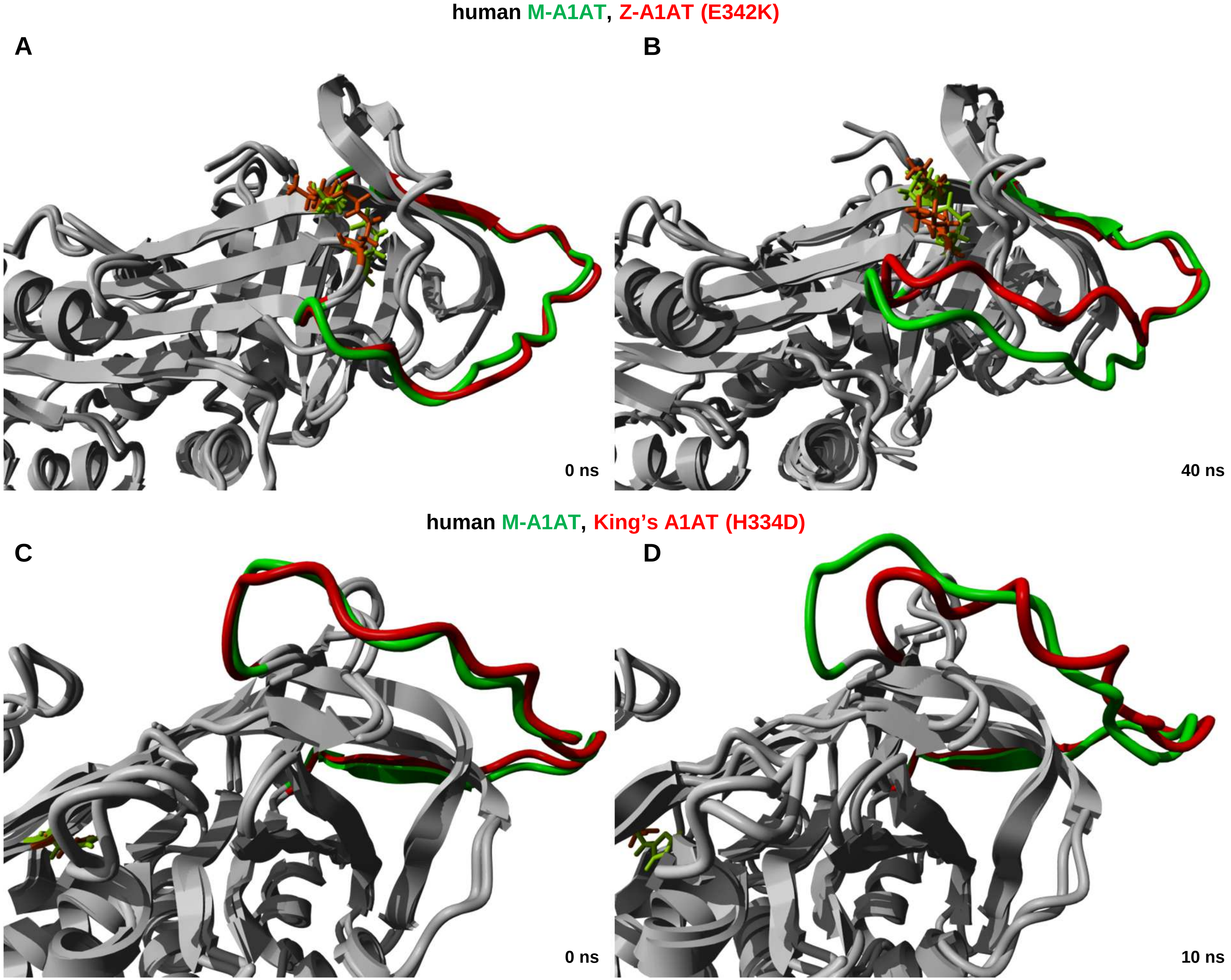

Supplementary Figure S2: Molecular dynamics simulation of human A1AT. (A-B) RCLs of wild type (green) and E342K mutant (red) A1AT at the start and after 40 ns of MD simulation. Distortion of RCL was found after 10 ns MD simulation when introducing the H334D point mutation **(C-D).** Amino acid residues involved in non-covalent bond formation at mutation sites are highlighted in light green (wt) and orange (mut).

Figure S3

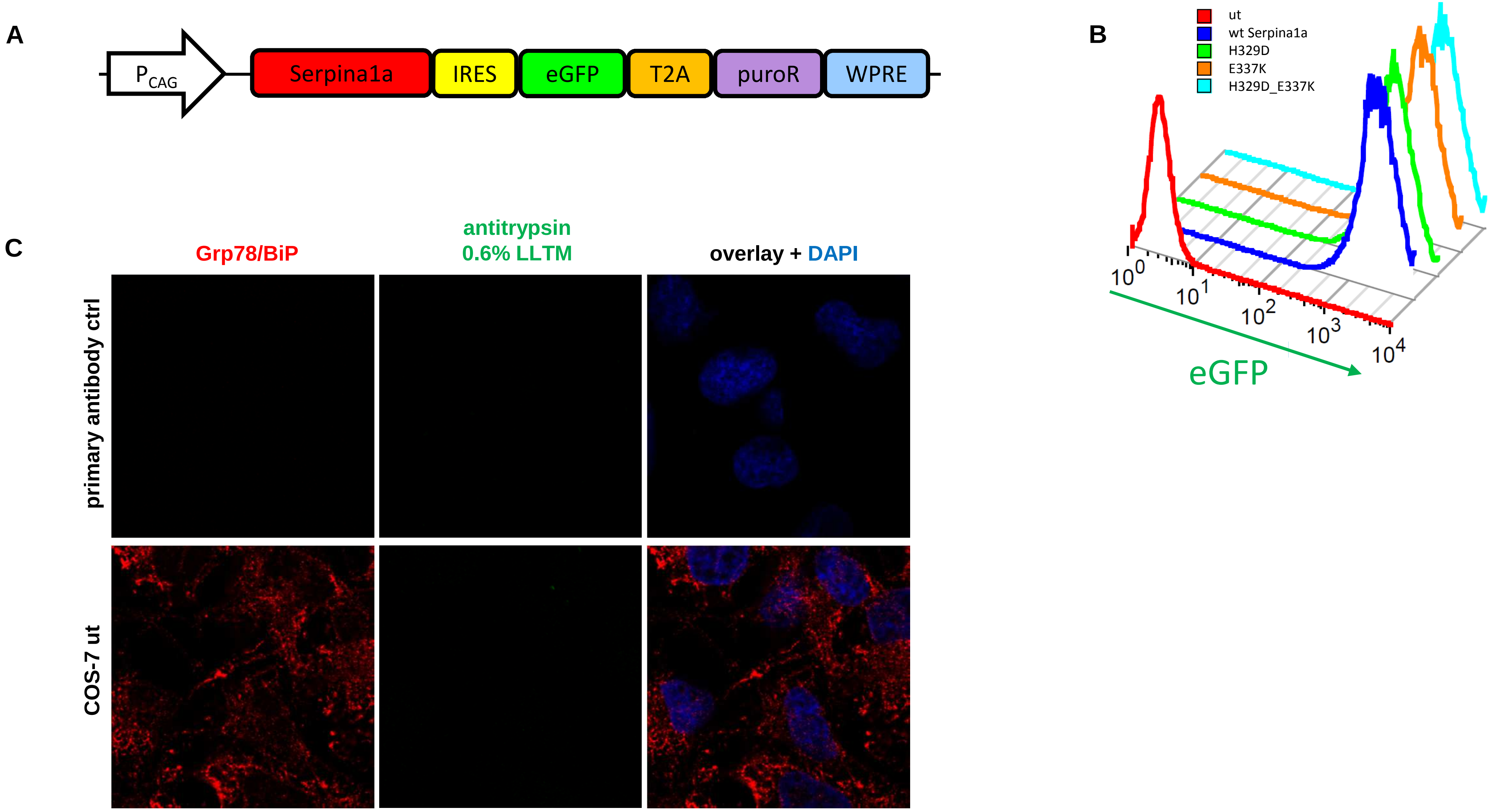

Supplementary Figure S3: Vector map and FACS evaluation of lentiviral vectors and laser microscopy controls. (A) Schematic of lentiviral vector for overexpression of wild type and mutant Serpina1a **(B)** Histograms of COS-7 cells transduced with lentiviral vector overexpressing mouse Serpina1a and eGFP (blue: wt Serpina1a; green, orange and light blue: Serpina1a mutants; red: untransduced; ut). **(C)** Top row: Laser confocal microscopy of COS-7 cells expressing Serpina1a stained and analyzed by using the same protocol as main Fig 2A, without addition of primary antibodies. Bottom row: untransduced COS-7 cells. Scale bar: 50 µm.

Figure S4

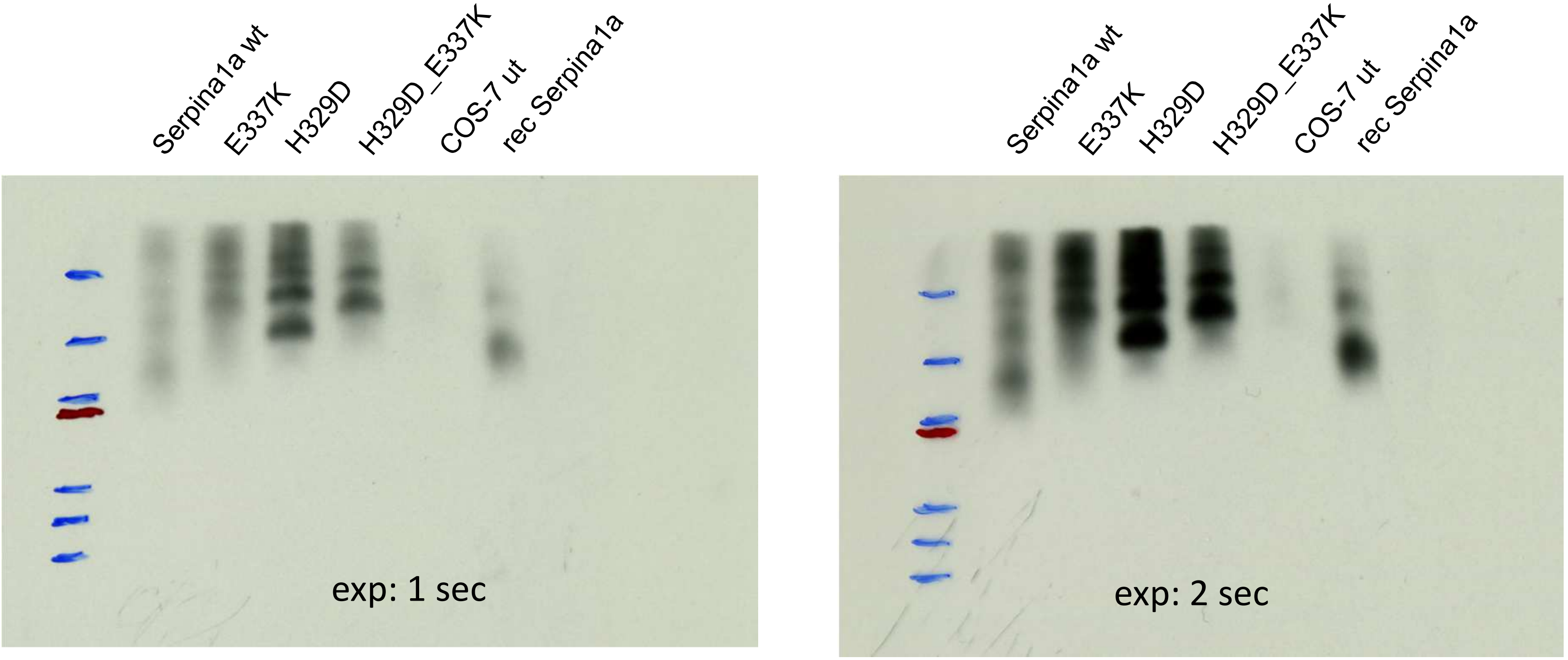

Supplementary Figure S4: Full and uncropped images of native western blot, main Figure 3B. Two different exposure times from native western blot of Figure 3B are shown as uncropped images. Protein ladder was included on the left for internal control purposes but does not represent actual band sizes, as isoelectric point influences position in a native gel.

Figure S5

A

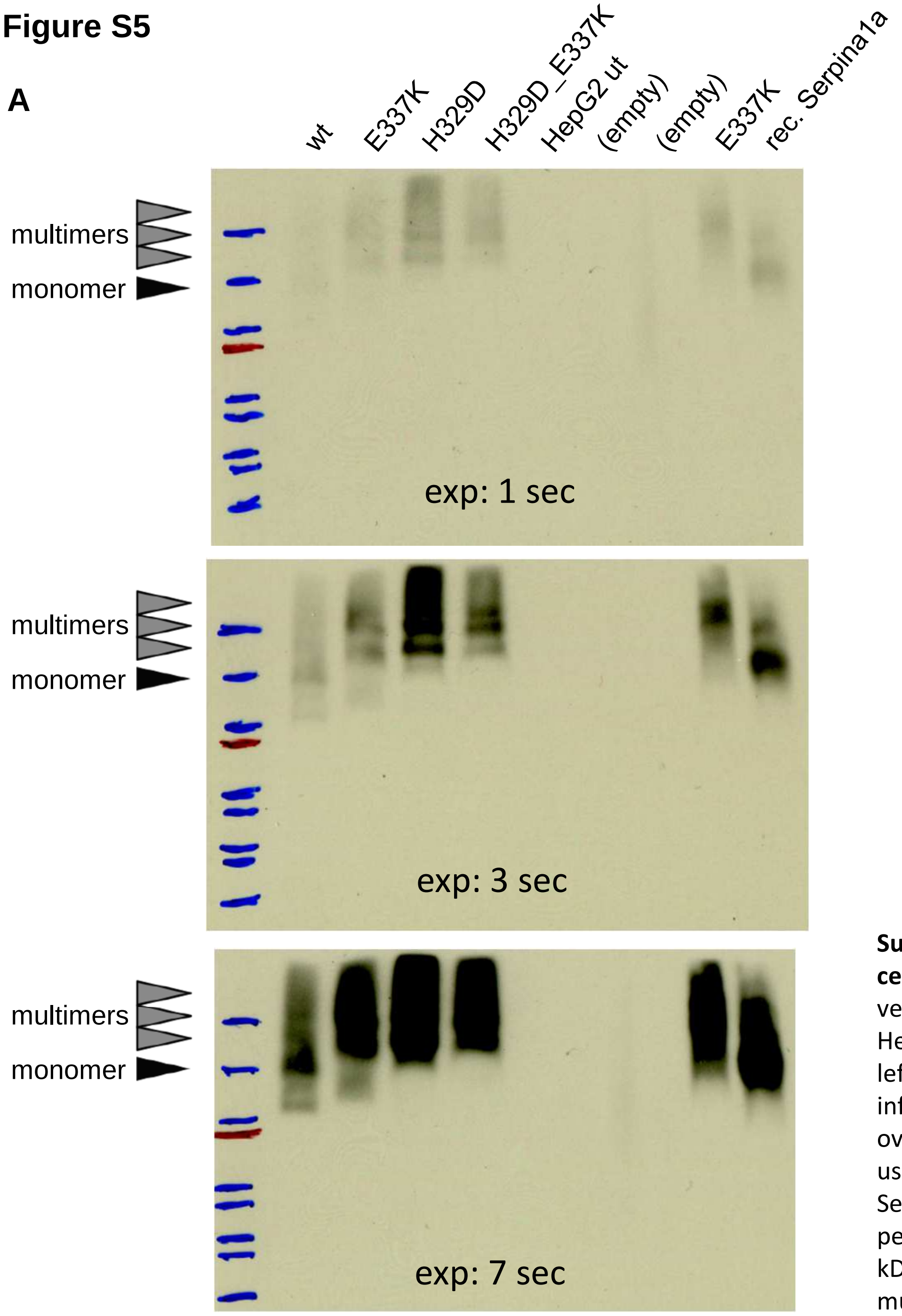

B

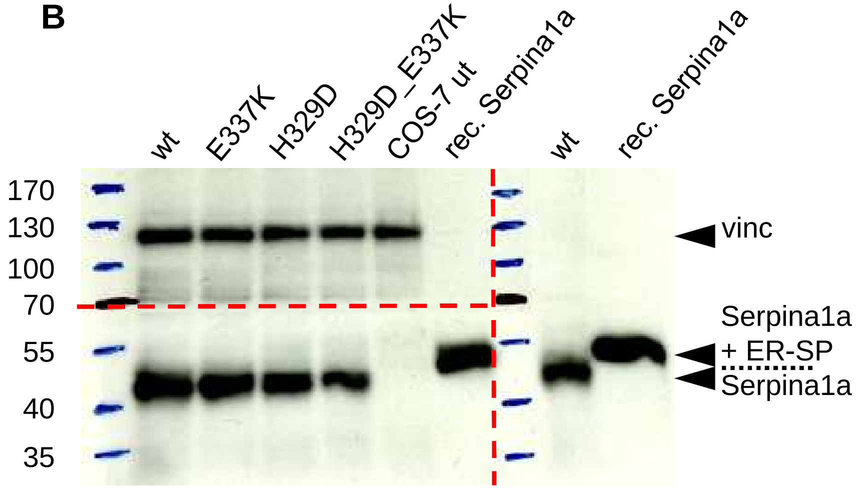

C

Isoelectric points of Serpina1a mutants

wt	5.44
E337K	5.58
H329D	5.32
H329D_E337K	5.45

Supplementary Figure S5: Western blots of mutant Serpina1a overexpressed in HepG2 and COS-7 cells. (A) Native western Blot of human hepatocarcinoma HepG2 cell lysates transduced with lentiviral vectors overexpressing wild type or mutant Serpina1a. Recombinant Serpina1a and untransduced HepG2 cells served as positive and negative controls, respectively. Protein ladder was included on the left for internal control purposes but does not represent actual band sizes, as isoelectric point influences the position in a native gel. **(B)** SDS-PAGE western blot of cell lysates from COS-7 cells overexpressing wild type or mutant Serpina1a. Blot was cut at red dotted lines and upper left part was used for detection of vinculin, whereas lower left and entire right part were used for detection of Serpina1a. Higher band size of Serpina1a isolated from recombinant bacteria results from ER signal peptide (ER-SP). Marker was traced from original blot and numbers on the left indicate band sized in kDa **(C)** Isoelectric points of wt mouse Serpina1a compared to E337K, H329D and H329D_E337K mutants were calculated using ExPASy compute pI/Mw tool (https://web.expasy.org/compute_pi/).

Figure S6

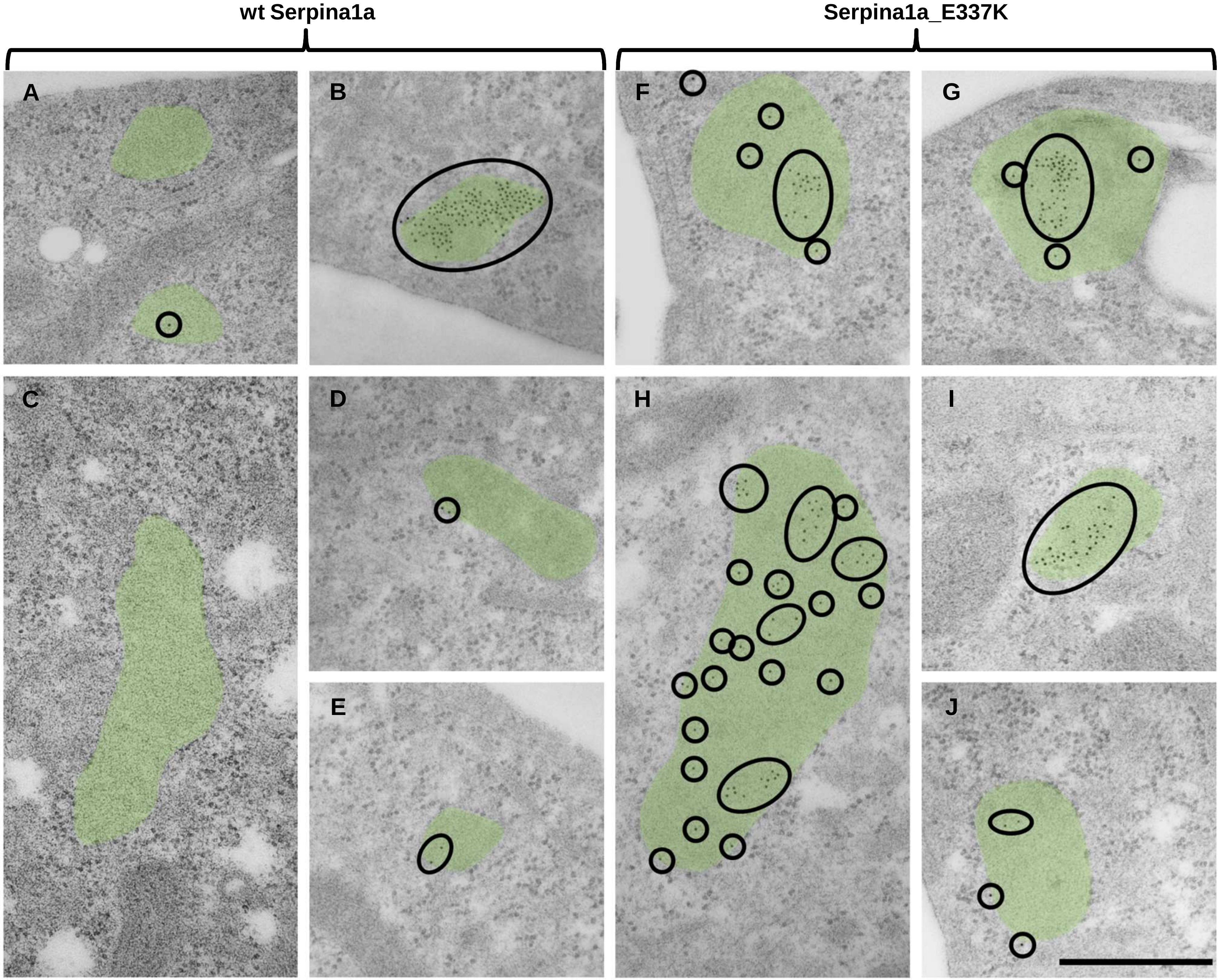

Supplementary Figure S6: Immuno-electron microscopy of Serpina1a-expressing COS-7 cells. Immunogold labelling of Serpina1a on sections of wt Serpina1a (**A-E**) and E337K mutant (**F-J**). Cellular compartments resembling dilated ER are highlighted in green. Positive antibody reaction is visualized by 10 nm gold particles (encircled by black lines for better visualization). Electron dense dots in the cytoplasm represent ribosomes. Scale bar: 500 nm.

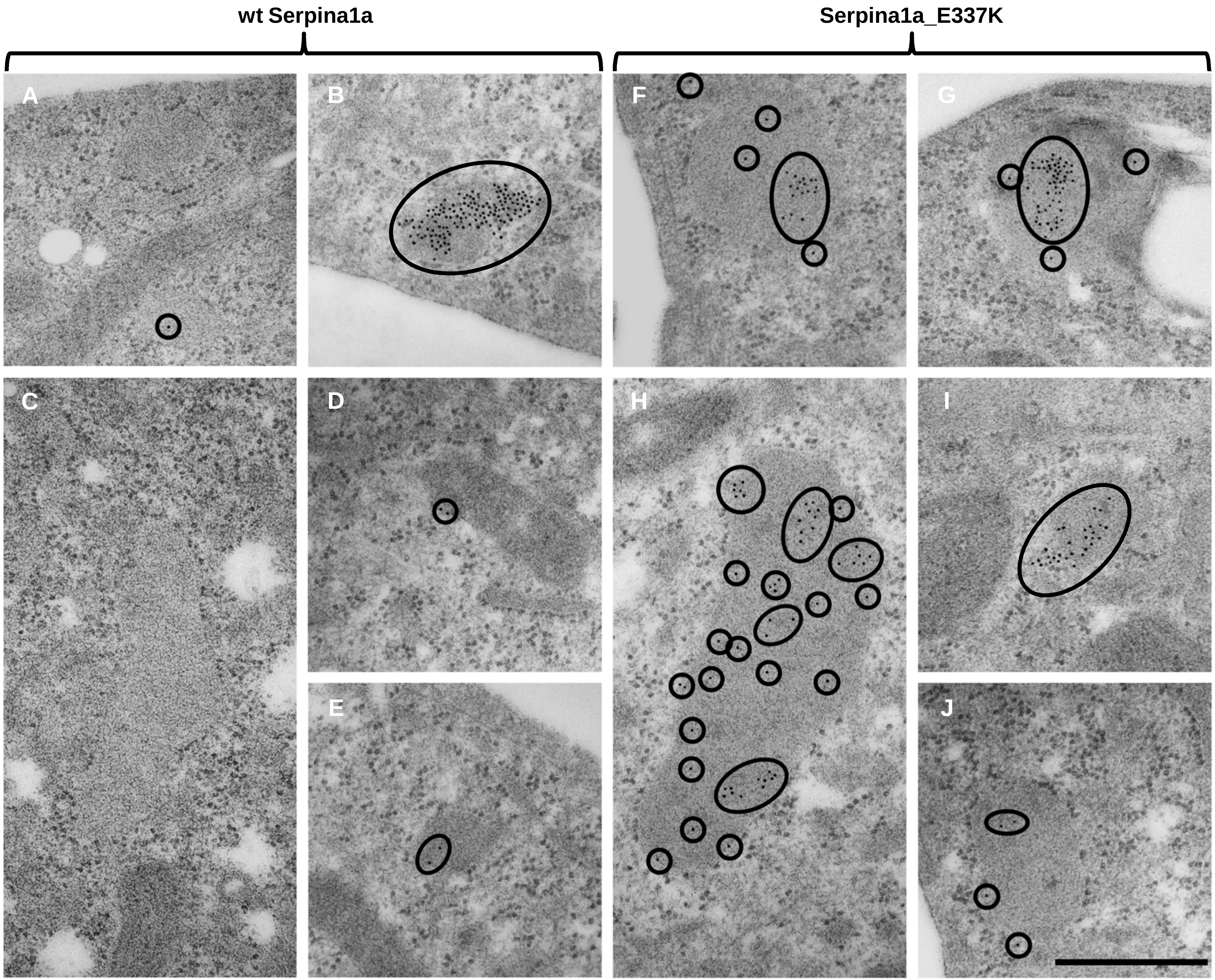

High contrast version of Figure S6 without colorization of ER.

Figure S7A

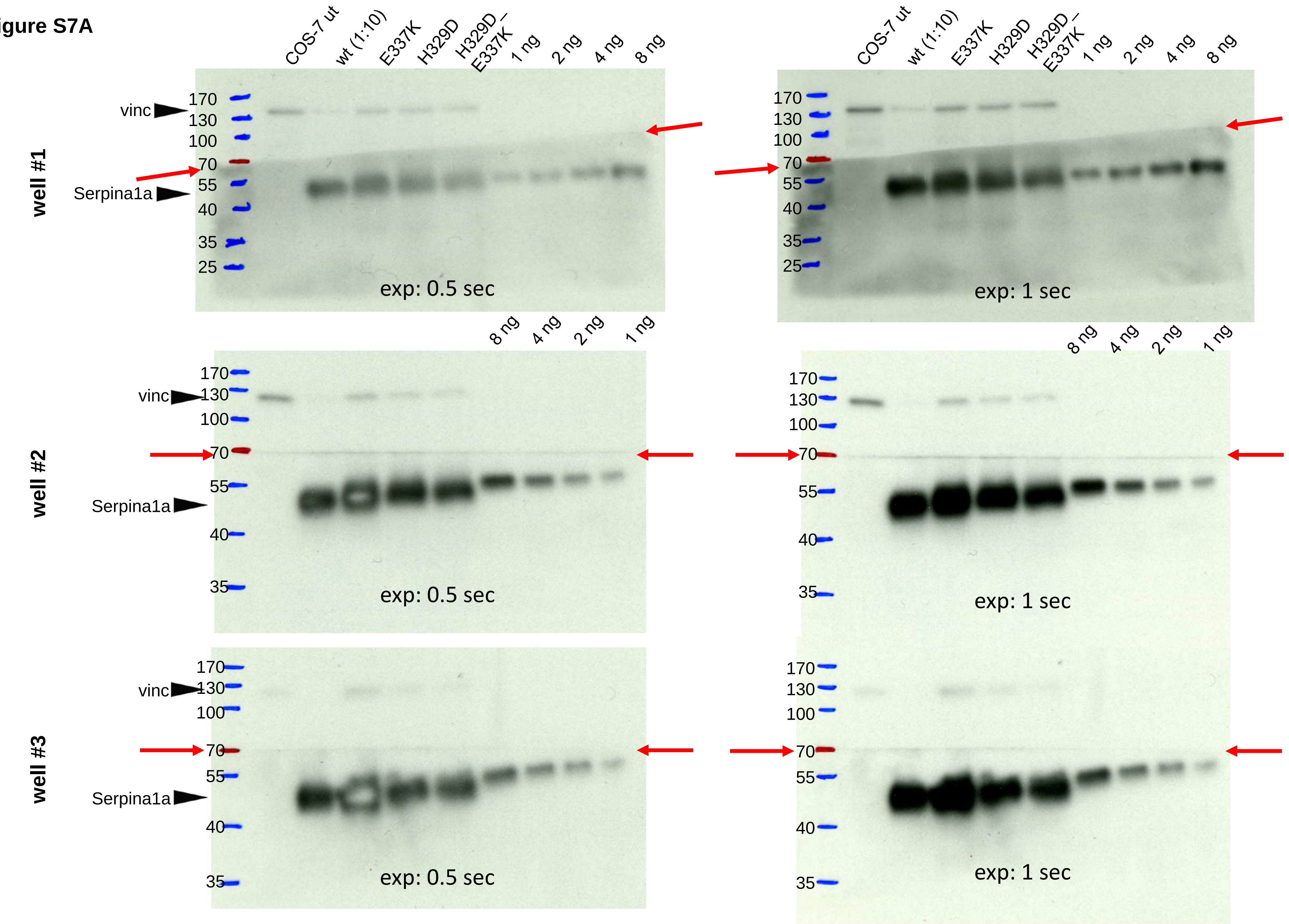

COS-7 ut
wt (1:10)
E337K
H329D
H329D_E337K
1 ng
2 ng
4 ng
8 ng
well #1
well #2
well #3
vinc
Serpina1a
170
130
100
70
55
40
35
25
exp: 0.5 sec
exp: 1 sec

Figure S7B

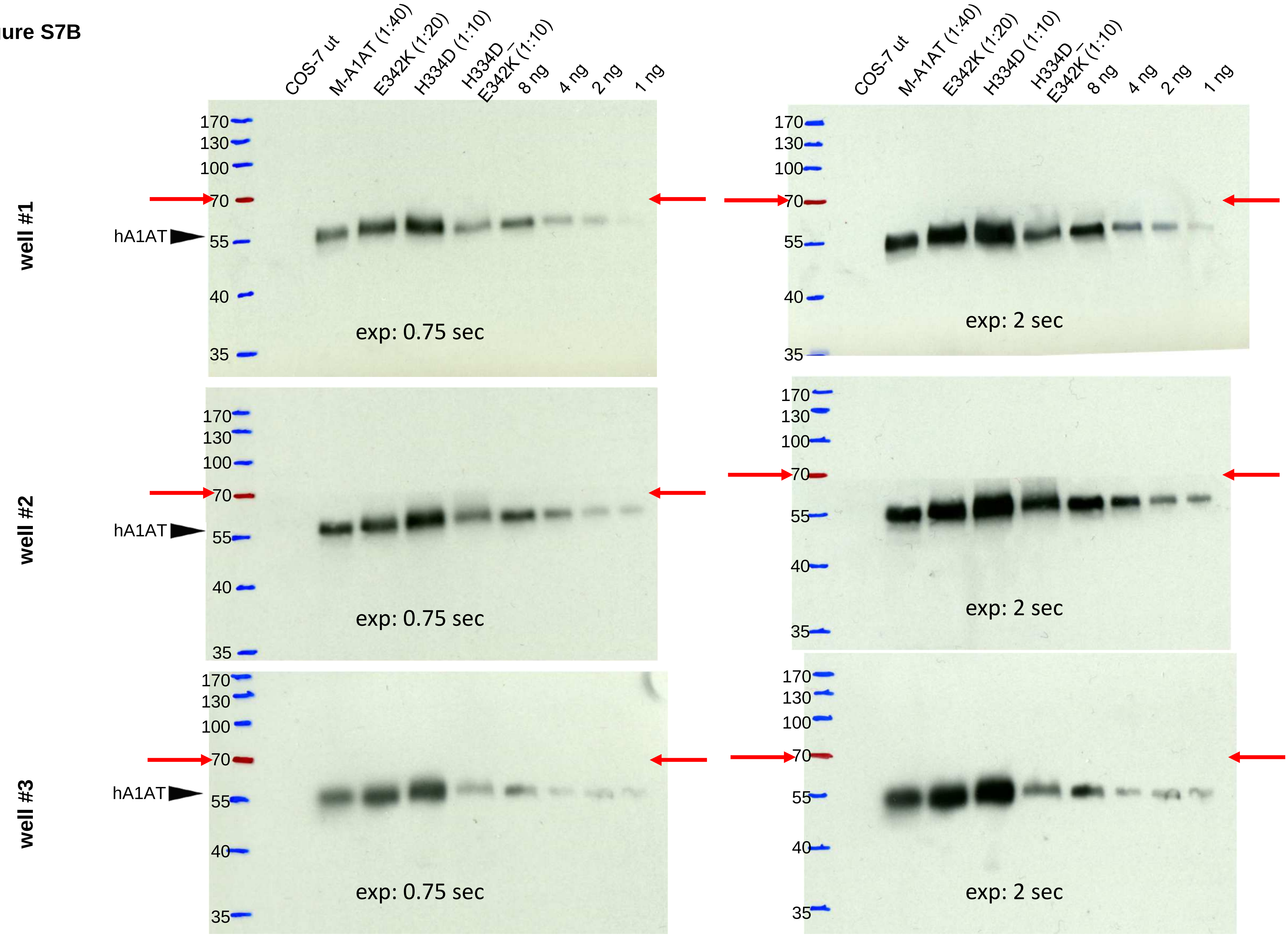

COS-7 ut
M-A1AT (1:40)
E342K (1:20)
H334D (1:10)
H334D_E342K (1:10)
8 ng
4 ng
2 ng
1 ng
well #1
well #2
well #3
hA1AT
170
130
100
70
55
40
35
exp: 0.75 sec
exp: 2 sec

Figure S7C-D

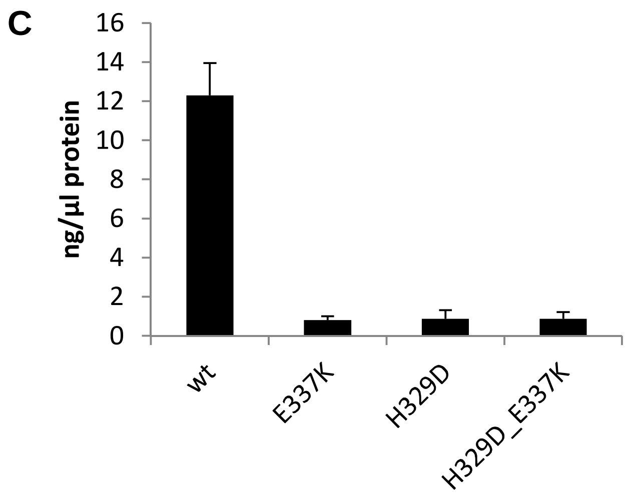

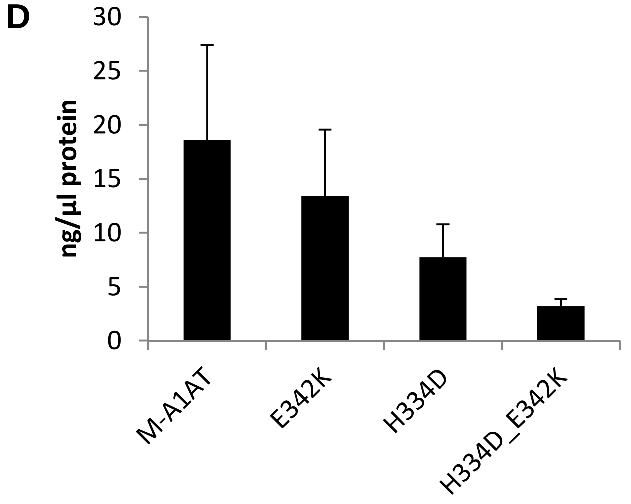

Supplementary Figure S7: SDS-PAGE western blot analysis and quantification of secreted Serpina1a and A1AT in supernatants from transgenic COS-7 cells. Western blot analysis of supernatants from 3 individual collections of wild type and mutant Serpina1a **(A)** or A1AT **(B)** expressing COS-7 cells. 1, 2, 4 or 8 ng of recombinant Serpina1a or A1AT were analyzed in the respective right four lanes. Note that supernatants from wt Serpina1a and from human A1AT-expressing cells were diluted according to the values in brackets. Blots were cut at red 70 kDa bands, as indicated by red arrows. Each blot is shown with two different exposure times; Serpina1a: 0.5 sec and 1 sec; A1AT: 0.75 sec and 2 sec. Supernatants from Serpina1a-expressing cells were additionally analyzed for vinculin for internal control purposes and full blots including these bands are shown here for completeness. However, vinculin is not a secreted protein and therefore no conclusions are drawn thereof. **(C, D)** Absolute protein concentrations in supernatants calculated after densitometry analysis of western blots. Exposure time of 0.5 sec was analyzed for Serpina1a and exposure time of 0.75 sec was analyzed for A1AT. Error bars represent positive standard deviation of 3 individually collected supernatant samples. A detailed statistical analysis using one-way ANOVA can be found in Supplementary Table 2.

Supplementary Table 1

Cloning primers

primer name	sequence
DOM-1 NsiI rev	ACCATGCATTCATTTATGTGTGGGATCTACC
D329 for	CTCTCCGGAATCACAGAGGAAAATGCTCCCCTGAAGCTCAGCCAGGCTGTG**g**ATAAGGCTGTGCTGACCATCGATGAGACAGGAACAG
K337 for	CTCTCCGGAATCACAGAGGAAAATGCTCCCCTGAAGCTCAGCCAGGCTGTGCATAAGGCTGTGCTGACCATCGAT**a**AGACAGGAACAG
D329 K337 for	CTCTCCGGAATCACAGAGGAAAATGCTCCCCTGAAGCTCAGCCAGGCTGTG**g**ATAAGGCTGTGCTGACCATCGAT**a**AGACAGGAACAG
hA1AT cDNA AgeI for	ATAACCGGTATGCCGTCTTCTGTCTCGTGGG
hA1AT cDNA NsiI rev	TTTATGCATTTATTTTTGGGTGGGATTCACCACTTTTCCC
H334D AvaI rev	GACCTCGGGGGGGATAGACATGGGTATGGCCTCTAAAAACATGGCCCCAGCAGCTTCAGTCCCTTTCTCGTCGATGGTCAGCACAGCCTTAT**c**CACGGCCTTGG
E342K AvaI rev	GACCTCGGGGGGGATAGACATGGGTATGGCCTCTAAAAACATGGCCCCAGCAGCTTCAGTCCCTTTCT**t**GTCGATGGTCAGCACAGCCTTATGCACGGCCTTGG
H334D E342K AvaI rev	GACCTCGGGGGGGATAGACATGGGTATGGCCTCTAAAAACATGGCCCCAGCAGCTTCAGTCCCTTTCT**t**GTCGATGGTCAGCACAGCCTTAT**c**CACGGCCTTGG

Supplementary Table 1: Sequences of cloning primers. Primers for molecular cloning of human and mouse antitrypsin-overexpression constructs were employed as described in methods section. Mutant bases for site-directed mutagenesis are indicated in red lower case letters.

Supplementary Table 2

Supplementary Table 2: Statistical analysis for Figures 4A-B and Supplementary Figure S6C-D. One-way ANOVA with Tukey's post test at 95% CI was performed using GraphPad PRISM

Figure 4A (NE inh. human), one-way ANOVA with Tukey's post test at 95% CI

P value	< 0.0001
P value summary	****
Are means signif. different? (P < 0.05)	Yes
Number of groups	5
F	170.1
R square	0.9855

ANOVA Table	SS	df	MS
Treatment (between columns)	4.22E+08	4	1.06E+08
Residual (within columns)	6.21E+06	10	620808
Total	4.29E+08	14	

Tukey's Multiple Comparison Test	Mean Diff.	q	Significant? P < 0.05?	P Summary	95% CI of diff
COS-7 ut vs M-A1AT	13596	29.89	Yes	***	11479 to 15713
COS-7 ut vs E342K	8389	18.44	Yes	***	6272 to 10506
COS-7 ut vs H334D	1977	4.345	No	ns	-140.4 to 4094
COS-7 ut vs H334D_E342K	381.3	0.8383	No	ns	-1736 to 2498
M-A1AT vs E342K	-5207	11.45	Yes	***	-7324 to -3090
M-A1AT vs H334D	-11620	25.54	Yes	***	-13737 to -9503
M-A1AT vs H334D_E342K	-13215	29.05	Yes	***	-15332 to -11098
E342K vs H334D	-6413	14.1	Yes	***	-8530 to -4296
E342K vs H334D_E342K	-8008	17.6	Yes	***	-10125 to -5891
H334D vs H334D_E342K	-1595	3.507	No	ns	-3712 to 521.8

Figure S6D (WB human), one-way ANOVA with Tukey's post test at 95% CI

P value	0.0433
P value summary	*
Are means signif. different? (P < 0.05)	Yes
Number of groups	4
F	4.327
R square	0.6187

ANOVA Table	SS	df	MS
Treatment (between columns)	405.6	3	135.2
Residual (within columns)	250	8	31.25
Total	655.6	11	

Tukey's Multiple Comparison Test	Mean Diff.	q	Significant? P < 0.05?	P Summary	95% CI of diff
M-A1AT vs E342K	5.227	1.619	No	ns	-9.390 to 19.84
M-A1AT vs H334D	10.89	3.375	No	ns	-3.724 to 25.51
M-A1AT vs H334D_E342K	15.43	4.781	Yes	*	0.8133 to 30.05
E342K vs H334D	5.666	1.756	No	ns	-8.951 to 20.28
E342K vs H334D_E342K	10.2	3.162	No	ns	-4.413 to 24.82
H334D vs H334D_E342K	4.538	1.406	No	ns	-10.08 to 19.15

Figure 4B (NE inh. mouse), one-way ANOVA with Tukey's post test at 95% CI

P value	< 0.0001
P value summary	****
Are means signif. different? (P < 0.05)	Yes
Number of groups	5
F	114.6
R square	0.9787

ANOVA Table	SS	df	MS
Treatment (between columns)	3.01E+08	4	7.53E+07
Residual (within columns)	6.57E+06	10	657444
Total	3.08E+08	14	

Tukey's Multiple Comparison Test	Mean Diff.	q	Significant? P < 0.05?	P Summary	95% CI of diff
COS-7 ut vs wt	8641	18.46	Yes	***	6462 to 10819
COS-7 ut vs E337K	9345	19.96	Yes	***	7166 to 11523
COS-7 ut vs H329D	917	1.959	No	ns	-1262 to 3096
COS-7 ut vs H329D_E337K	-1079	2.305	No	ns	-3258 to 1100
wt vs E337K	704	1.504	No	ns	-1475 to 2883
wt vs H329D	-7724	16.5	Yes	***	-9902 to -5545
wt vs H329D_E337K	-9720	20.76	Yes	***	-11898 to -7541
E337K vs H329D	-8428	18	Yes	***	-10606 to -6249
E337K vs H329D_E337K	-10424	22.27	Yes	***	-12602 to -8245
H329D vs H329D_E337K	-1996	4.264	No	ns	-4175 to 182.7

Figure S6C (WB mouse), one-way ANOVA with Tukey's post test at 95% CI

P value	< 0.0001
P value summary	****
Are means signif. different? (P < 0.05)	Yes
Number of groups	4
F	127.7
R square	0.9795

ANOVA Table	SS	df	MS
Treatment (between columns)	294.6	3	98.2
Residual (within columns)	6.152	8	0.7691
Total	300.8	11	

Tukey's Multiple Comparison Test	Mean Diff.	q	Significant? P < 0.05?	P Summary	95% CI of diff
wt vs E337K	11.49	22.68	Yes	***	9.192 to 13.78
wt vs H329D	11.42	22.56	Yes	***	9.131 to 13.72
wt vs H329D_E337K	11.42	22.55	Yes	***	9.125 to 13.71
E337K vs H329D	-0.06126	0.121	No	ns	-2.354 to 2.232
E337K vs H329D_E337K	-0.06749	0.1333	No	ns	-2.361 to 2.226
H329D vs H329D_E337K	-0.00623	0.0123	No	ns	-2.299 to 2.287
